# Euxanthone inhibits traumatic spinal cord injury via anti-oxidative stress and suppression of p38 and PI3K/Akt signaling pathway in a rat model

**DOI:** 10.1515/tnsci-2021-0012

**Published:** 2021-03-17

**Authors:** Rubin Yao, Lirong Ren, Shiyong Wang, Ming Zhang, Kaishun Yang

**Affiliations:** Department of Spine Surgery, The First Affiliated Hospital of Dali University, Dali City, No. 32 Carlsberg Avenue, Yunnan, 671000, China

**Keywords:** euxanthone, spinal cord injury, NF-κB, PI3K, Akt

## Abstract

**Background:**

Owing to neurite promoting, antioxidant and anti-inflammatory effects of Euxanthone (Eux), the investigation was aimed to probe the neuroprotective efficacy of Eux against traumatic spinal cord injury (t-SCI) in rats and whether Eux can improve neuropathic function in t-SCI.

**Method:**

Sprague-Dawley (SD) rats were randomized in – Sham, t-SCI, Eux30, and Eux60 (t-SCI + 30 and 60 mg/kg respectively). Animals with compression force-induced t-SCI were subjected to estimation of locomotor functions. Spinal cord water content and Evans blue (EB) effusion were determined for quantifying edema and intactness of the spinal cord. Oxidative stress and immunochemical markers were quantified by ELISA and western blotting.

**Results:**

Findings revealed that Eux60 group animals had greater Basso, Beattie, and Bresnahan (BBB) and (incline plane test) IPT score indicating improved locomotor functions. There was a reduction in the spinal edema and water content after Eux treatment, together with lowering of oxidative stress markers. The expression of IL-6, IL-12, IL-1β, caspase-3, RANKL, TLR4, NF-κB, p-38, PI3K, and Akt in spinal cord tissues of t-SCI-induced rats was lowered after Eux treatment.

**Conclusion:**

Overall, the investigation advocates that Eux attenuates t-SCI and associated inflammation, oxidative damage, and resulting apoptosis via modulation of TLR4/NF-κB/p38 and PI3K/Akt signaling cascade.

## 1 Introduction

Among the most grievous events worldwide is the traumatic spinal cord injury (t-SCI) that leads to serious perpetual neurological malfunction and neuronal degeneracy [1]. Owing to direct or indirect damage to the spinal cord during t-SCI, the affected individual along with their family members may experience several challenges during daily life [2]. Developed countries have a high morbidity rate in comparison to developing countries [3].

The t-SCI may be originated from diverse direct and indirect injuries that may result in desensitization over the injured area, inflammation, and partial or complete limb dysfunctionality [4,5]. Several primary and secondary pathophysiological events are precipitated after the t-SCI episode. The primary events are provoked spontaneously, including tissue injury, axonal breakdown, and vascular disruption [6]. Associated secondary aspects resulted from primary events are delayed, which include series of biochemical, molecular, and cellular alterations, leading to oxidative stress, inflammation, and pain that may extend from hours to weeks [7,8,9,10]. The t-SCI originated inflammatory responses and associated cells affect the functionality and responses of organs including kidney, lungs, and liver [11].

Insufficient oxygen level in the mitochondria under oxidative stress results in elevated reactive oxygen species (ROS) levels [12,13]. Both generation and breakdown of ROS are maintained during normal situations; however, under an event of t-SCI, the ROS quantum is raised [14]. This elevated ROS quantum leads to several damaging effects including lipid peroxidation, oxidation of proteins, and damage to the DNA. Consecutively, excessive ROS can trigger specific pathways that result in cell death [15]. Excessive ROS is known to result in cell apoptosis by stimulating the p38 MAPK signaling cascade, which is documented to be critically involved in the development of t-SCI [16,17]. Neuroapoptosis is among the major factors that contribute to the weak prognosis of t-SCI [18].

One of the important kinases, protein kinase B (Akt), is involved in the regulation of growth, differentiation, and apoptosis of cells. Akt is triggered by its localization onto the interior of the cell membrane surface followed by conversion into its phosphorylated derivative, p-Akt [19]. Activation of phosphatidylinositol 3-kinase (PI3K) into p-PI3K by phosphorylation of the -OH group on the third position of inositol moiety subsequently translocates the serine/threonine-protein kinase [20]. Earlier investigations indicate that Akt stimulation imparts neuroprotection by reducing the neuroapoptosis, lowering the formation of oxygen-free radical, and attenuating the inflammation during t-SCI episode [19,21].

Reports reveal that the mitogen-activated protein kinase (MAPK) family has three subfamily signaling cascades, namely p38 MAPK, extracellular regulated protein kinase (ERK1/2), and c-Jun N-terminal kinase (JNK). On activation by lipopolysaccharides, the MAPK signaling cascades generate several inflammatory mediators through intricate intracellular signal transduction, resulting in inflammatory responses [22]. Enhanced phosphorylation is resulted by triggering of ERK1/2, JNK, and MAPK p38 signaling cascade [23].

Xanthones are long known to exert several biological effects. Pharmacological activities mediated by xanthones include anti-inflammatory, anticancer, anti-thrombotic, and anti-microbial properties [24,25,26,27,28]. Euxanthone, a xanthone derivative isolated from the plant Polygala Caudata, has been reported to promote neurite outgrowth [29,30]. In the ovarian cancer cell model, Eux has been found to induce autophagy, which is attributed to pSTAT3/Bcl-2 modulation [31]. Eux imparts protection to human umbilical vein endothelial cells (HUVECs) damaged due to oxidative and inflammatory stress mediated by oxidized low-density lipoprotein [32]. Both *in vivo* and *in vitro*, Eux exerts a neuroprotective effect against neurotoxicity induced by Aβ_1–42_. Eux remarkably attenuates memory and spatial learning impairment caused by Aβ_1–42_ and apoptosis and reverted neuroapoptosis in hippocampal and cortical regions. Eux also reduces oxidative stress and ROS generation caused by Aβ_1–42_ in the rat model [33]. There has been no reported data to assess the therapeutic effect of Eux on t-SCI. Therefore, we investigated the therapeutic efficacy of Eux in t-SCI in the adult male Sprague-Dawley (SD) rat model. An attempt was made to explore whether Eux can attenuate neuronal apoptosis by reducing oxidative stress and modulating the p38 and Akt signaling pathway in rats induced with t-SCI.

## 2 Materials and methods

### 2.1 Animal model

Thirty-two male SD rats, weighing 220–250 g, procured from the central animal house of The First Affiliated Hospital of Dali University, were housed in a hygienic environment at 23 ± 3°C, 40–60% relative humidity, and exposed to 12 h light and 12 h dark, with unrestricted access to diet and water. The animals were shuffled into four groups (*n* = 8): Sham group, t-SCI group, Eux30 group (t-SCI + Eux 30 mg/kg), and Eux60 group (t-SCI + Eux 60 mg/kg).


**Ethical approval:** The research related to animals’ use has been complied with all the relevant national regulations and institutional policies for the care and use of animals.

### 2.2 Induction of t-SCI and Eux administration

The surgical intervention to induce t-SCI was carried out as reported earlier [34]. The rat was allowed to lay down and fixed in a supine posture on the operating platform. Initially, before the surgery, the skin hairs over the vertebral column of the rats were shaved and 1% pentobarbital (40 mg/kg) was injected intraperitoneally. An incision (20 mm) was made along the midline at the T10 vertebral foci. Lamina was exposed after the surgical incision and laminectomy of the T10 vertebral column was performed to reveal the spinal cord. Further, vascular clip (Kent Scientific, Torrington, USA) graduated to exert 30 g force was employed to clamp the spinal cord for 30 s, without damaging the dura mater, thereby leading to compression-induced injury. The skin and musculature in the rats were layered and sutured in place carefully. The sham group rats were exposed to the entire surgical procedure except for compression by vascular clip (Kent Scientific, Torrington, USA). After surgery, the rats were injected intraperitoneally with 5 mL physiological saline solution for maintaining intraperitoneal volume and were maintained warm throughout the recovery process. Animals from group Eux30 (30 mg/kg) and Eux60 (60 mg/kg) received Eux diluted in physiological saline solution, once within 3 days for 10 weeks. Until the urinary activity was recovered, the rats were provided with bladder massage two times a day to ease urination.

### 2.3 Assessment of motor function

Basso, Beattie, and Bresnahan (BBB) [35] and inclined plane test (IPT) [36] scores were determined for the assessment of motor function, on 1st day, 3rd, 7th, and 10th week after t-SCI. The rats from all study groups were assessed for BBB score on a scale between 0, indicating complete non-functionality of hind limbs, to 21, indicating almost normal locomotor functionality. For estimation of IPT score, the locomotory axis of the animal body and the rigid inclined plane were aligned in the same direction, and gradually the angle of the plane concerning the horizontal was increased and recorded at which the rats could maintain themselves for 5 s. Both the investigations were conducted by individuals which were blind to animal clusters under study. The observations were noted for three separate experiments, and an average was considered as the final result.

### 2.4 Estimation of water content in the spinal cord

The spinal cord water content was estimated by employing the wet-dry method [37]. Spinal cord samples (15 mm length) were collected from the epicenter of the damage and wet weight was recorded without any delay. Further, these specimens were subjected to drying for 72 h at 95°C, followed by weighing to note the dry weight. The observer was blind to the spinal cord samples collected from the rats from experimental groups. The water content in the spinal cord was calculated using the following formula.

\text{Spinal}\hspace{.5em}\text{cord}\hspace{.5em}\text{water}\hspace{.5em}\text{content}\hspace{.5em}( \% )=\frac{\text{wet}\hspace{.5em}\text{weight}-\text{dry}\hspace{.5em}\text{weight}}{\text{wet}\hspace{.5em}\text{weight}}\times 100]

### 2.5 Blood spinal cord barrier leakage determination

The blood spinal cord barrier leakage was estimated by the extent of Evan’s blue (EB) effusion [38,39]. The EB was delivered post-anesthetization via the femoral vein. After one hour, 0.1 M physiological buffer saline was perfused transcardially and spinal cord samples (10 mm) were collected. The spinal cord samples were subjected to homogenization in 3 mL trichloroacetic acid (50%) and further cryocentrifuged (4°C) for 20 min at 12,000× *g*. Further, 1 mL of resulting supernatant was added to a blend of 1 mL of ethyl alcohol and trichloroacetic acid solution (1:3) and preserved overnight at 4°C. The samples were subjected to centrifugation for 30 min at 15,000× *g* at 4°C. The supernatant was analyzed at 680 nm emission wavelength and 620 nm excitation wavelength using a microplate reader (Victor Nivo Multimode plate reader, PerkinElmer, Shanghai, China). The resulting values were converted to EB content (in mg) using the standard curve. The values were estimated thrice and an average value was finalized as the result, in terms of EB content (in mg) in a unit weight of tissues (in g).

### 2.6 Estimation of caspase-3 activity, inflammatory cytokines, and oxidative stress markers

The rats were subjected to anesthesia by injecting 10% chloral hydrate (3.5 mg/kg) intraperitoneally, followed by the collection of peripheral blood from the orbital cavity, and subjecting it to centrifugation at 2,000× *g* at 4°C for 10 min for the separation of serum. ELISA kit and a fluorescence microplate reader (Victor Nivo Multimode plate reader, PerkinElmer, Shanghai, China) were used to estimate the caspase-3 activity (Catalogue No. C1116) (Beyotime Institute of Biotechnology, Haimen, China) at 405 nm. ELISA kits using a fluorescence microplate reader at 450 nm were used to quantify using inflammatory cytokines – Interleukin (IL)-6 (Catalogue No. PI328), IL-12 (Catalogue No. H010), and IL-1b (Catalogue No. PI303) (Beyotime Institute of Biotechnology, Haimen, China), and markers of oxidative stress – malondialdehyde (MDA) (Catalogue No. S0131), glutathione (GSH) (Catalogue No. S0052), and glutathione peroxidase (GSH-PX) (Catalogue No. S0058) (Beyotime Institute of Biotechnology, Haimen, China).

### 2.7 Western blot analysis

The spinal cord tissues were added to a blend of cold radioimmunoprecipitation assay (RIPA) lysis buffer and phenylmethylsulfonyl fluoride (100:1) for 30 min and subjected to a cooling centrifuge at 2,000× *g* for 12 min. The protein content in the resulting supernatant was estimated using a bicinchoninic acid protein assay kit. Around 50 mg of proteins was collected and separated using 10% sodium dodecyl sulfate-polyacrylamide gel electrophoresis (SDS-PAGE) and moved over polyvinylidene difluoride membranes (Merck Life Science (Shanghai) Co., Ltd. Shanghai, China). The membrane was then occluded for an hour at 37°C using a mixture of tris-buffer saline and 5% skimmed milk. The membranes were then incubated for 12 h at 4°C with primary antibodies purchased from Bio-rad Laboratories, Shanghai, China, namely – Receptor activator of nuclear factor-κB ligand (RANKL) (MCA5971), toll-like receptor 4(TLR4) (AHP1822), Nuclear factor (NF)-κB (VPA00015), phosphorylated (p)-NF-κB (AHP1342), PI3K (AHP2224), Akt (VMA00253) and GAPDH (HCA272), p38 (AHP2410), and p-p38 (#9211, Cell Signaling Technology, Shanghai, China). The membranes were then rinsed thrice with a blend of tris-buffer saline and 0.1% Tween 20 for 20 min and subjected to incubation with horseradish peroxidase (HRP) conjugated anti-mouse IgG secondary antibody (#7076, 1:5,000) at 37°C for an hour, followed by visualization by chemiluminescence and quantification using Western blot detection system (#7072, Cell Signaling Technology, Shanghai, China) and Image J version 3.0 analysis software (www.imagej.nih.gov/ij), respectively.

### 2.8 Statistical treatment of data

Statistical operations were performed using Microsoft Excel in Windows operating system. Data between groups were compared using one-way ANOVA, and subsequently by Duncan’s multiple range test. The results are presented as the mean ± standard error. The *p*-value of less than 0.01 and 0.05 was designated as statistically significant.

## 3 Results

### 3.1 Eux enhanced the BBB and IPT score in t-SCI-induced rats

**Figure 1:**
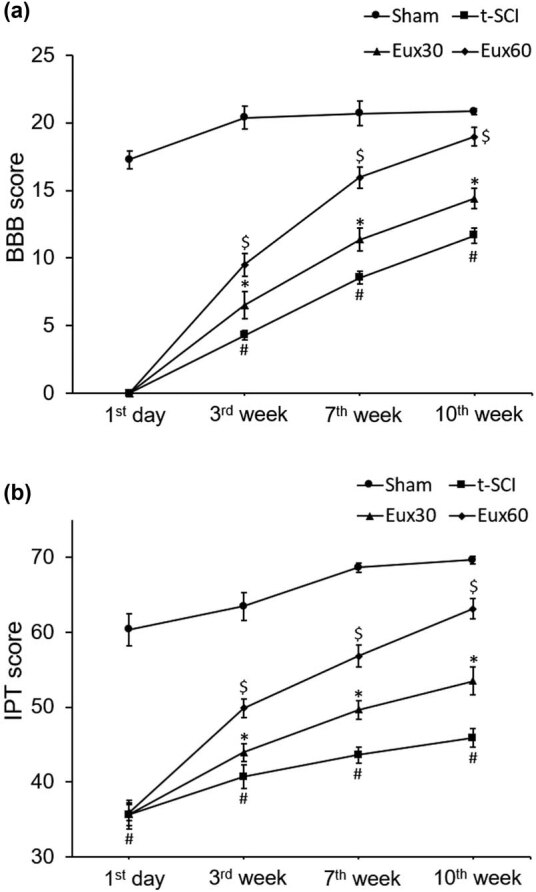
Effect of Euxanthone on the BBB scores and IP scores in t-SCI-induced rats after 1st day, 3rd, 7^th^, and 10th week of investigation. Euxanthone effects on (a) BBB scores and (b) IPT scores in t-SCI-induced rats. The data are represented as the mean value ± standard error of mean. ^#^ *p* < 0.01 relative to sham group, *p < 0.05 relative to t-SCI model group, and ^$^ *p* < 0.05 relative to t-SCI model group and Eux30 study group. t-SCI, traumatic spinal cord injury; Eux30 and Eux60, Euxanthone 30 and 60 mg/kg, respectively, in t-SCI-induced rats; BBB, Basso, Beattie and Bresnahan, and IPT, inclined plane test.

The BBB and IPT score were determined to assess the motor functions after induction of t-SCI in rats. Observations revealed that BBB and IPT scores were slightly lowered after surgical intervention, which eventually returned to normal values with the progression of time. The BBB and IPT scores were reduced to almost 0 after surgical treatment and subsequently increased in further days in the Sham group and not in the t-SCI group. In t-SCI group animals, the BBB and IPT scores indicated reduced recovery, compared to the Sham group and Eux treated groups. Animals from groups Eux30 and Eux60 indicated a significant (*p* < 0.05) increase in the BBB and IPT scores in a dose-dependent manner after the 7th and 10th week, compared to the t-SCI group that was left untreated (Figure 1a and b).

### 3.2 Eux lowered the spinal cord water content and EB effusion in t-SCI-induced rats

**Figure 2:**
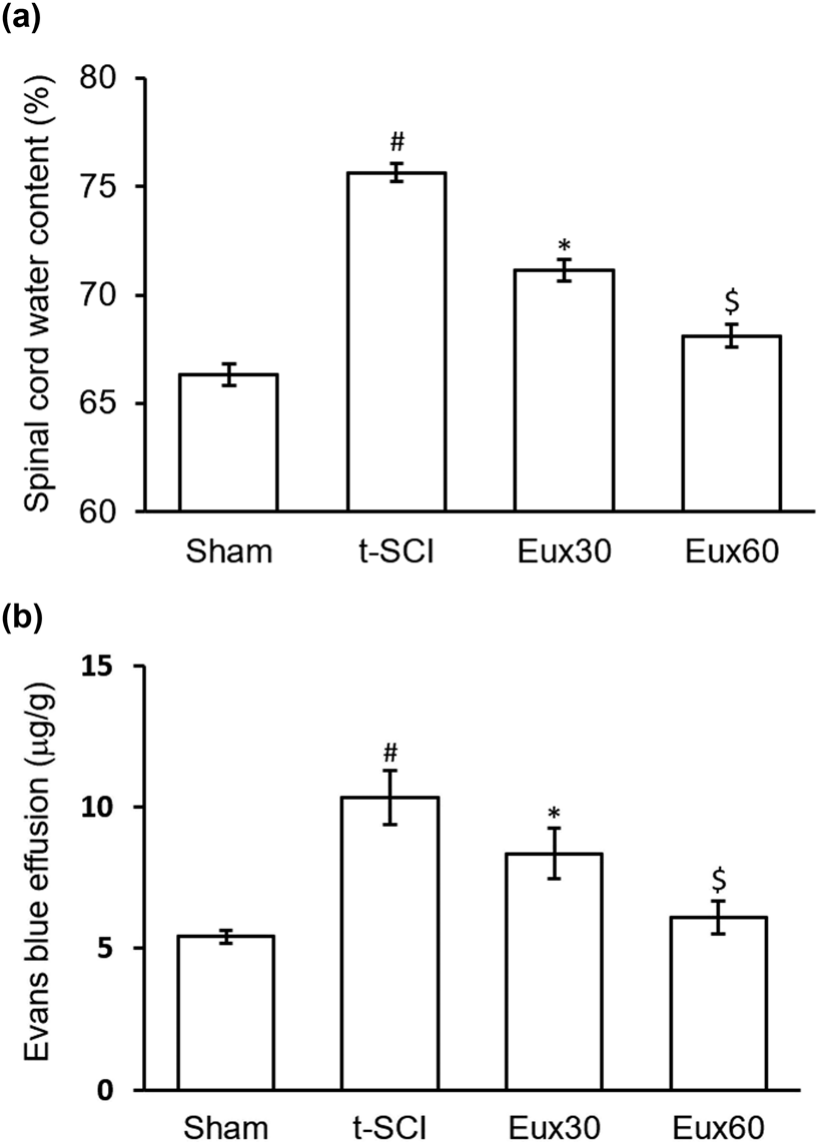
Effect of Euxanthone on the spinal cord water content and Evans blue effusion in the spinal cord of t-SCI-induced rats. Euxanthone effects on (a) spinal cord water content and (b) Evans blue effusion in the spinal cord of t-SCI-induced rats. The data are represented as the mean value ± standard error of mean. ^#^ *p* < 0.01 relative to sham group, *p < 0.05 relative to t-SCI model group, and ^$^ *p* < 0.05 relative to t-SCI model group and Eux30 study group. t-SCI, traumatic spinal cord injury; Eux30 and Eux60, Euxanthone 30 and 60 mg/kg, respectively, in t-SCI-induced rats.

Qualitative and quantitative investigation of blood spinal cord barrier leakage after t-SCI was performed, respectively, by measurement of EB effusion and determination of spinal cord water content. Both were significantly (*p* < 0.01) enhanced in t-SCI-induced animals compared to the animals from the Sham group. However, there was a remarkable (*p* < 0.05) decrease in the spinal cord water content and EB effusion in animals induced with t-SCI and subsequently treated with Eux. Animals from the Eux60 group, receiving the highest dose of Eux, indicated a maximum reduction in the spinal cord water content and EB effusion, almost close to the values observed in Sham group animals (Figure 2a and b).

### 3.3 Eux attenuated inflammatory cytokines in t-SCI-induced rats

**Figure 3:**
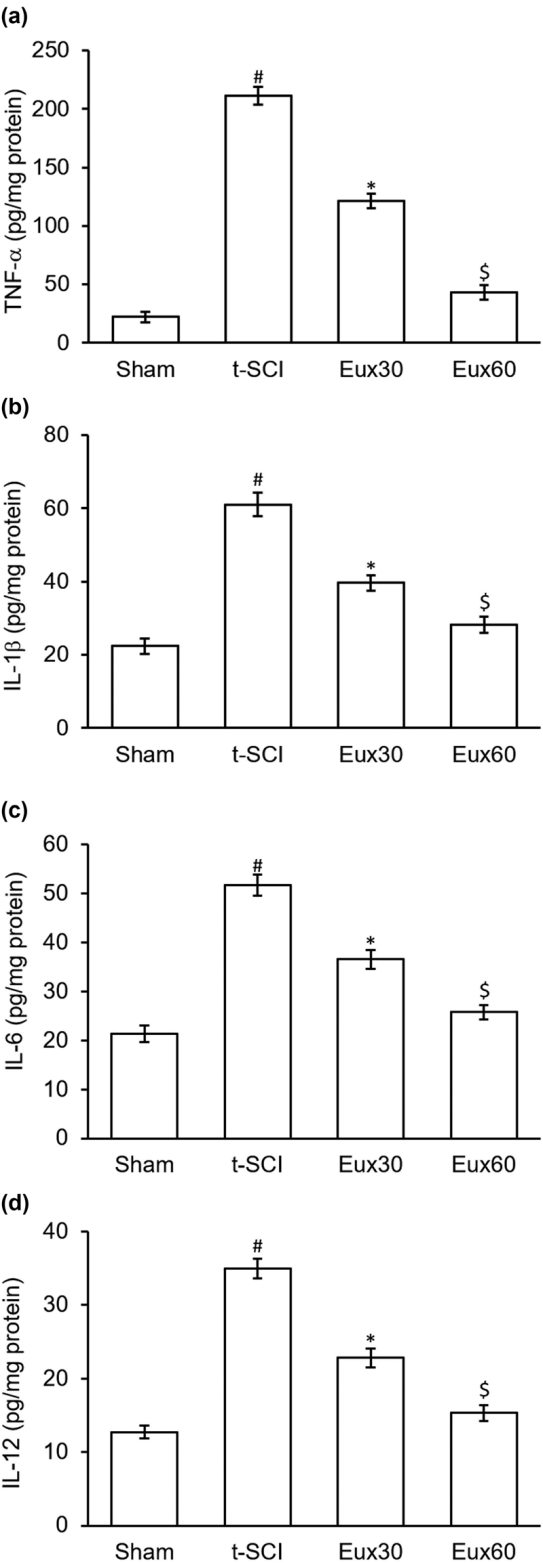
Effect of Euxanthone on inflammatory cytokines in t-SCI-induced rats. Euxanthone effects on (a) TNF-α, (b) IL-1β, (c) IL-6, and (d) IL-12 activities. The data are represented as the mean value ± standard error of mean. ^#^ *p* < 0.01 relative to sham group, *p < 0.05 relative to t-SCI model group, and ^$^ *p* < 0.05 relative to t-SCI model group and Eux30 study group. t-SCI, traumatic spinal cord injury; Eux30 and Eux60, Euxanthone 30 and 60 mg/kg, respectively, in t-SCI-induced rats; TNF, tumor necrosis factor; IL, interleukin.

Rats from t-SCI groups exhibited significant (*p* < 0.01) enhancement in the TNF-α, IL-6, IL-12, and IL-1β levels, compared to that in Sham group rats. However, Eux treatment significantly (*p* < 0.05) lowered the inflammatory cytokines- TNF-α, IL-6, IL-12, and IL-1β levels in spinal cord aliquots in comparison to the t-SCI-induced rats that were not treated with Eux. A marked increase in the attenuation of inflammatory cytokines was observed in animals that received a higher dose of Eux after the t-SCI event (Figure 3a–d).

### 3.4 Eux reduced the oxidative stress in t-SCI-induced rats

**Figure 4:**
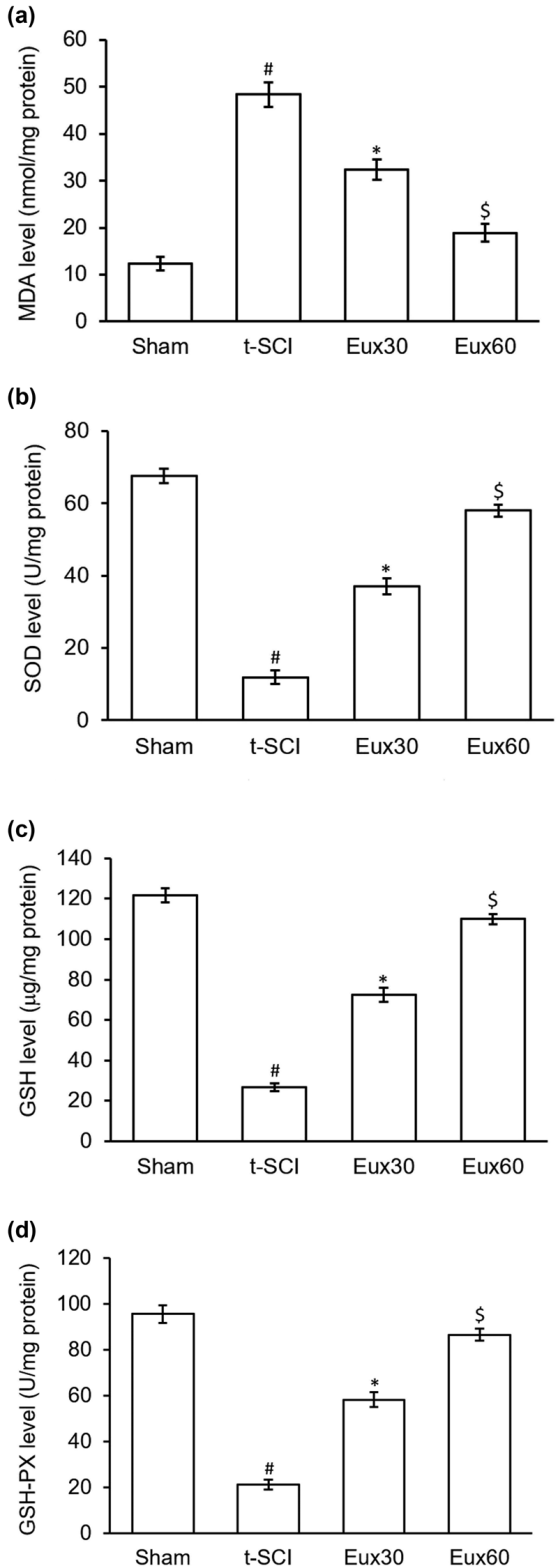
Effect of Euxanthone on levels of oxidative stress markers in t-SCI-induced rats. Euxanthone effects on (a) MDA, (b) SOD, (c) GSH, and (d) GSH-PX. The data are represented as the mean value ± standard error of mean. ^#^ *p* < 0.01 relative to sham group, *p < 0.05 relative to t-SCI model group, and ^$^ *p* < 0.05 relative to t-SCI model group and Eux30 study group. t-SCI, traumatic spinal cord injury; Eux30 and Eux60, Euxanthone 30 and 60 mg/kg, respectively, in t-SCI-induced rats; MDA, malondialdehyde; SOD, superoxide dismutase; GSH, glutathione; PX, peroxidase.

In comparison to the Sham group rats, the t-SCI-induced rats revealed significant (*p* < 0.01) enhancement in the MDA level and reduction in the levels of SOD, GSH, and GSH-PX. However, animals induced with t-SCI and treated with Eux exhibited marked lowering in the MDA activity and elevation in the activity of SOD, GSH, and GSH-PX activity. As revealed in Figure 4a–d, a significantly stronger antioxidant effect was seen in animals that received a higher dose of Eux that is 60 mg/kg (Figure 4a–d).

### 3.5 Eux suppressed the enhanced expression of RANKL in t-SCI-induced rats

**Figure 5:**
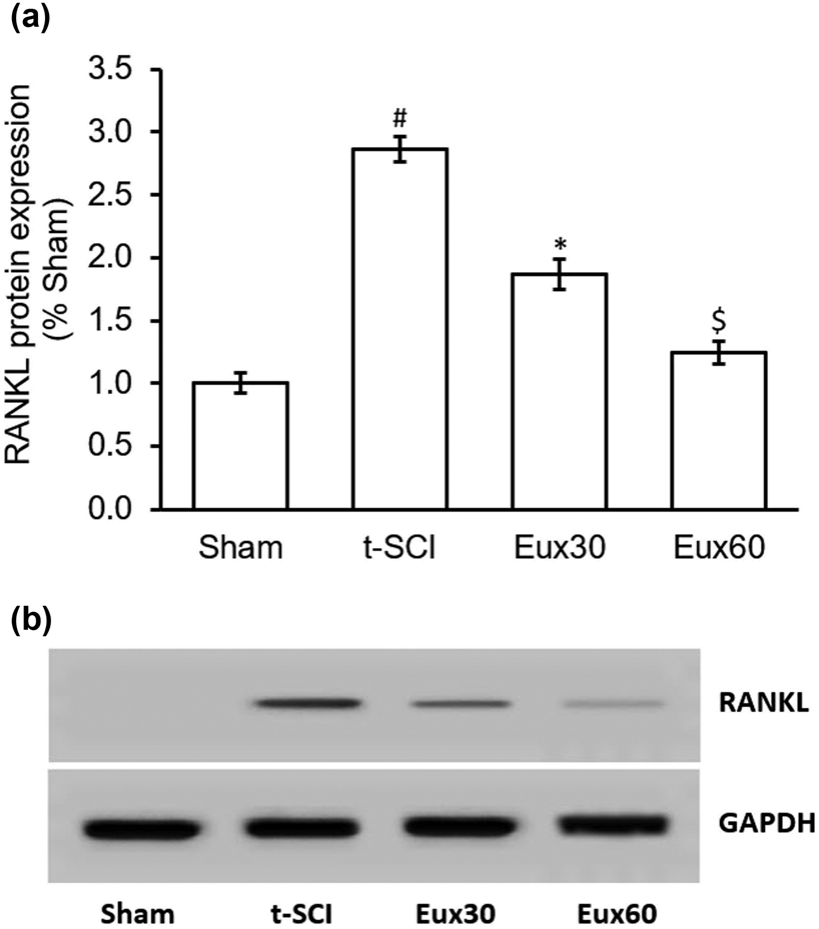
Effect of Euxanthone on RANKL protein expression in t-SCI-induced rats. (a) Quantification and (b) representative Western blot images of the effect of Euxanthone on RANKL protein expression. GAPDH served as an internal control. The data are represented as the mean value ± standard error of mean. ^#^ *p* < 0.01 relative to sham group, *p < 0.05 relative to t-SCI model group, and ^$^ *p* < 0.05 relative to t-SCI model group and Eux30 study group. t-SCI, traumatic spinal cord injury; Eux30 and Eux60, Euxanthone 30 and 60 mg/kg, respectively, in t-SCI-induced rats; RANKL, receptor activator of nuclear factor κB ligand.

The expression of RANKL in t-SCI-induced rats was estimated using Western blotting. There was a significantly (*p* < 0.01) higher RANKL protein expression in rats exposed to t-SCI, compared to the Sham group. As indicated in Figure 5a and b, rats from group Eux30 and Eux60, which were induced with t-SCI and treated with Eux30 and 60 mg/kg, revealed a significant (*p* < 0.05) reduction in RANKL protein expression when compared to the t-SCI-induced and untreated rats (Figure 5a and b).

### 3.6 Eux reduced the p-p38 expression in t-SCI-induced rats

**Figure 6:**
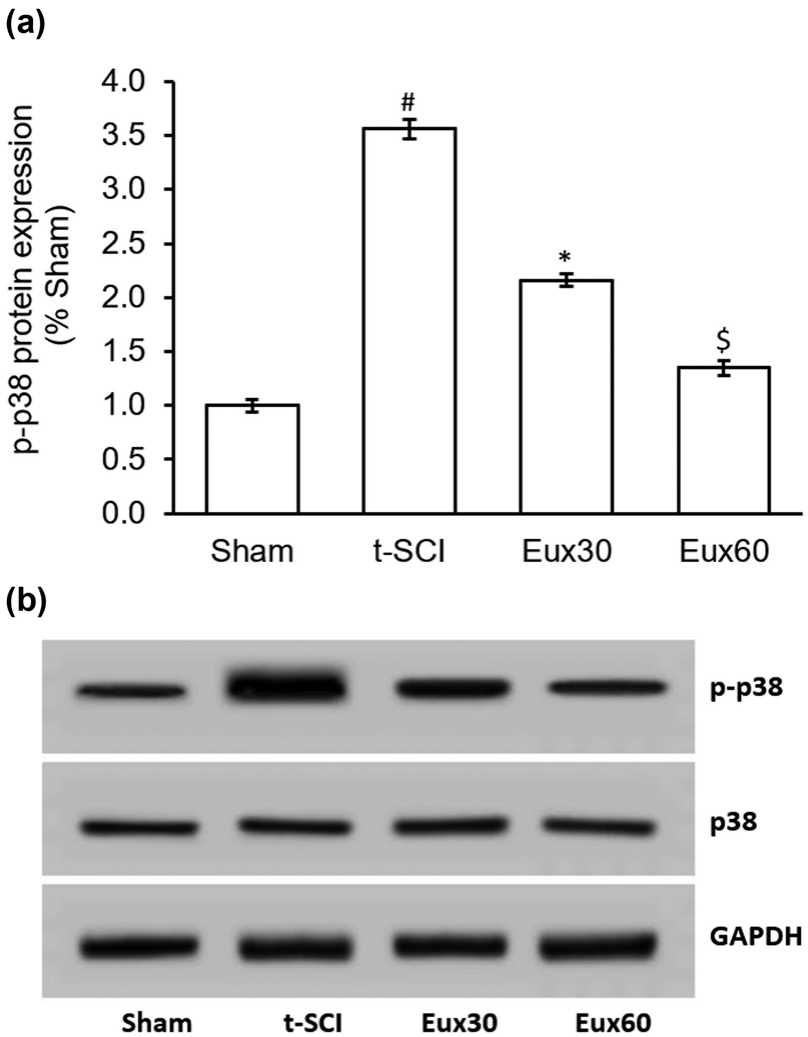
Effect of Euxanthone on p-p38 protein expression in t-SCI-induced rats. (a) Quantification and (b) representative Western blot images of the effect of Euxanthone on p-p38 protein expression. GAPDH served as an internal control. The data are represented as the mean value ± standard error of mean. ^#^ *p* < 0.01 relative to sham group, *p < 0.05 relative to t-SCI model group, and ^$^ *p* < 0.05 relative to t-SCI model group and Eux30 study group. t-SCI, traumatic spinal cord injury; Eux30 and Eux60, Euxanthone 30 and 60 mg/kg, respectively, in t-SCI-induced rats; p, phosphorylated.

The expression of p-p38 was remarkably (*p* < 0.01) enhanced in rats that were induced with t-SCI than in the rats from the Sham group. Rats from groups that were induced with t-SCI and then treated with Eux exhibited a significantly reduced expression of p-p38, in comparison to the rats that were subjected to t-SCI and left untreated. As observed in Figure 6a and b, among the Eux administered animals, those provided with a higher dose of Eux exhibited enhanced suppression of p-p38 protein expression (Figure 6a and b).

### 3.7 Eux attenuated the expression of inflammatory proteins in t-SCI-induced rats

**Figure 7:**
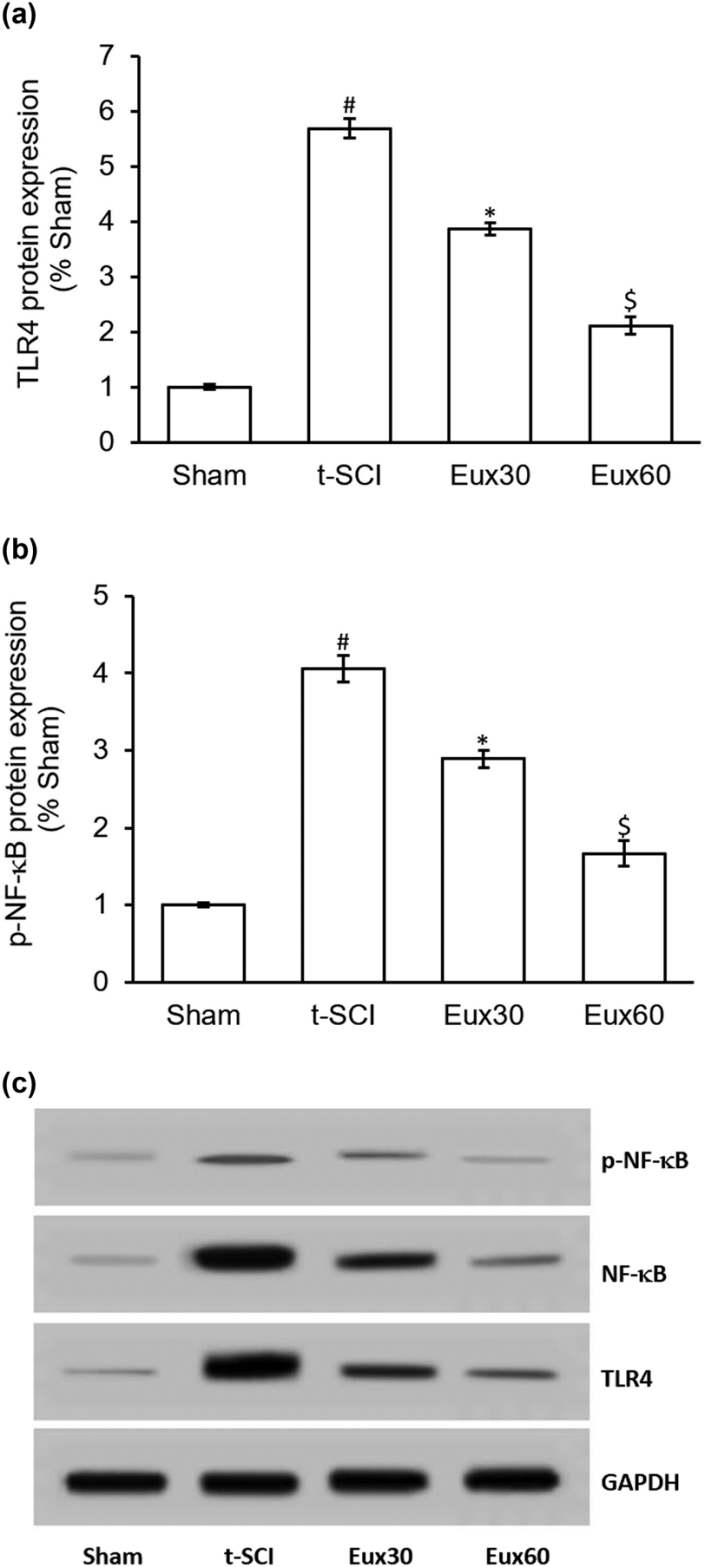
Effect of Euxanthone on TLR4/NF-κB protein expression in t-SCI-induced rats. (a) Quantification of TLR4 protein expression. (b) Representative Western blot images of the effects of Euxanthone on TLR4/NF-κB protein expression. (c) Quantification of NF-κB protein expression. GAPDH served as an internal control. The data are represented as the mean value ± standard error of mean. ^#^ *p* < 0.01 relative to sham group, *p < 0.05 relative to t-SCI model group, and ^$^ *p* < 0.05 relative to t-SCI model group and Eux30 study group. t-SCI, traumatic spinal cord injury; Eux30 and Eux60, Euxanthone 30 and 60 mg/kg, respectively, in t-SCI-induced rats; TLR4, toll-like receptor 4; NF-κB, nuclear factor; p, phosphorylated.

Western blot was employed to explore the activity of Eux on the activity of TLR4/NF-κB in t-SCI-induced rats. A marked (*p* < 0.01) increase in the activity of TLR4 and p-NF-κB was recorded in t-SCI-induced rats, relative to the Sham group animals. However, Eux administration dramatically lowered the expression of TLR4 and p-NF-κB in animals subjected to t-SCI (Figure 7a–c). Comparatively, in the animals from the Eux untreated group and Eux30 group, the effect was remarkably (*p* < 0.05) higher in animals from the Eux60 group (Figure 7a–c).

### 3.8 Eux suppressed the caspase-3 activity in t-SCI-induced rats

**Figure 8:**
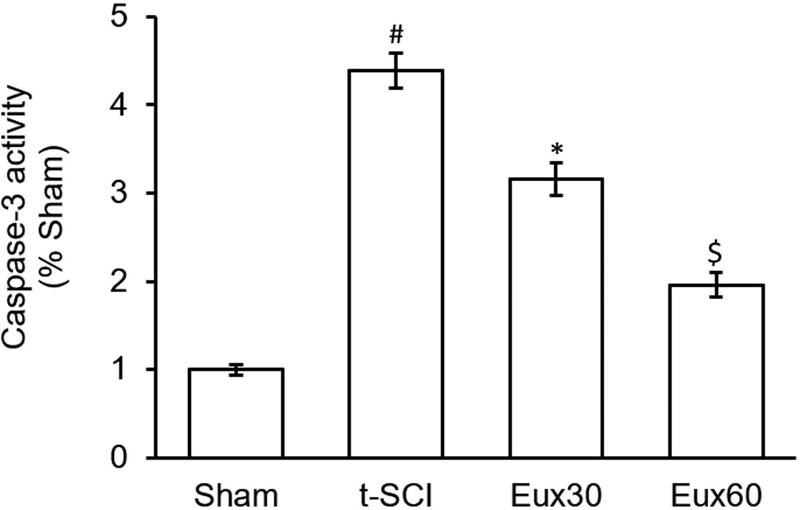
Effect of Euxanthone on caspase-3 activity in t-SCI-induced rats. The data are represented as the mean value ± standard error of mean. ^#^ *p* < 0.01 relative to sham group, *p < 0.05 relative to t-SCI model group, and ^$^ *p* < 0.05 relative to t-SCI model group and Eux30 study group. t-SCI, traumatic spinal cord injury; Eux30 and Eux60, Euxanthone 30 and 60 mg/kg, respectively, in t-SCI-induced rats.

As indicated in Figure 8, relative to the Sham group, a marked (*p* < 0.01) increase in the expression of caspase-3 was observed in rats from the t-SCI group. Treatment of rats with Eux (60 mg/kg) up to 10 weeks substantially (*p* < 0.05) suppressed the increased caspase-3 expression in rats from the t-SCI group, compared to Eux untreated t-SCI animals and Eux30 group animals (Figure 8a).

### 3.9 Eux normalized the altered PI3K/Akt activity in t-SCI-induced rats

**Figure 9:**
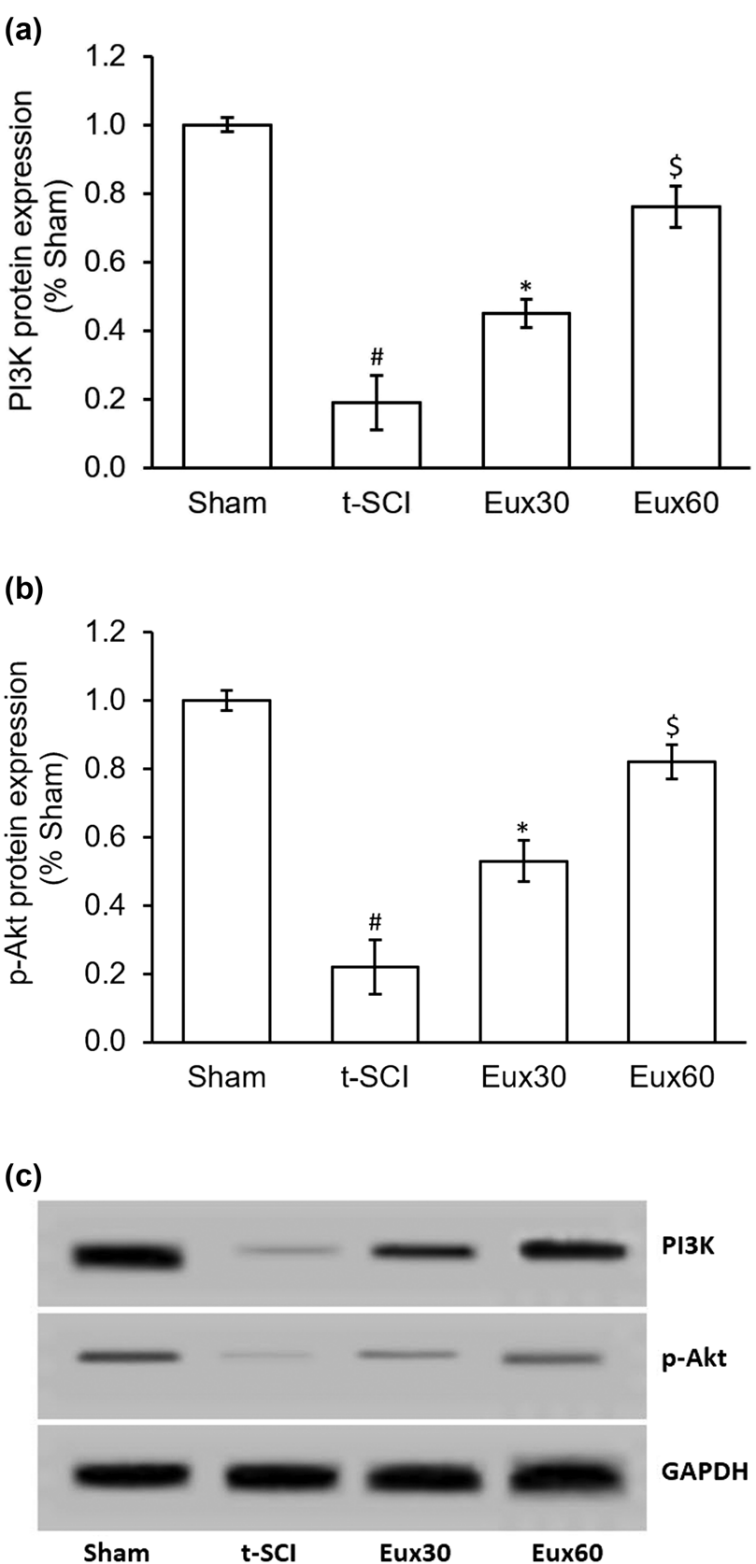
Effect of Euxanthone on PI3K/Akt protein expression in t-SCI-induced rats. (a) Quantification of PI3K protein expression. (b) Representative Western blot images of the effects of Euxanthone on PI3K/Akt protein expression. (c) Quantification of p-Akt protein expression. GAPDH served as an internal control. The data are represented as the mean value ± standard error of mean. ^#^ *p* < 0.01 relative to sham group, *p < 0.05 relative to t-SCI model group, and ^$^ *p* < 0.05 relative to t-SCI model group and Eux30 study group. t-SCI, traumatic spinal cord injury; Eux30 and Eux60, Euxanthone 30 and 60 mg/kg, respectively, in t-SCI-induced rats; Akt, protein kinase B; p, phosphorylated; PI3K, phosphatidylinositol 3-kinase.

To study the antiapoptotic efficacy of Eux in the t-SCI event, the expression of PI3K/Akt was estimated by Western blotting. As indicated in Figure 9a–c, when correlated to the Sham group rats, there was a significant (*p* < 0.01) reduction in the PI3K and p-Akt levels in t-SCI group rats. Administration of Eux in t-SCI-induced rats remarkably (*p* < 0.05) enhanced the activity of PI3K and p-Akt when correlated with the t-SCI group that was untreated with Eux. Also, a higher dose of Eux (60 mg/kg) indicated significant (*p* < 0.05) modulation in the PI3K/Akt expression, when compared to rats treated with a low dose of Eux (30 mg/kg) and the untreated t-SCI group rats (Figure 9a–c).

## 4 Discussion

SCI, as a result of physical and mechanical damage either by compression or structural breakdown of the spinal cord, leads to loss of sensation, stimulus, and locomotion and loss of sphincter control below the foci of damage [40]. There may be several causes of SCI, encompassing road accidents, injuries due to bullets and firearms, falls, and natural calamities [41]. Hence, SCI is regarded as an issue with high incidences of disability, thereby imposing a massive economic burden on the individual leading to poor quality of life [42]. Owing to these possible characteristics, SCI treatment has become a thrust area to be investigated for exploration of newer, efficacious, and safer molecules for its effective treatment. Earlier investigations have suggested that cell necrosis is the predominant landmark of SCI [43,44]. Also, reports claim that resulting necrosis and inflammation of the spinal cord associated neurons after primary mechanical damage are linked with apoptosis [43,45].

Our findings demonstrated that Eux potentially augmented the BBB and IPT score after induction of t-SCI in rats. A poor BBB and IPT score indicated that the rats were unable to make the normal movement of the limbs, which was owing to the spinal cord damage leading to loss of control over the voluntary movement of the limbs for locomotion. Higher BBB and IPT scores indicate proper and well-coordinated locomotion of the limbs. Lower BBB and IPT scores were observed in rats induced with t-SCI, whereas the scores were increased after Eux treatment. High Eux dose (60 mg/kg) indicated improvisation in the BBB and IPT scores marked by improved, coordinated, and stabilized locomotion in rats.

Marked pathological changes during t-SCI episode include edema and blood spinal cord barrier damage, which propose a challenge to its treatment [46]. Animals in which the t-SCI is induced by compression of the spinal cord using a clip indicate spinal cord distortion, damaged cell organelles, and breakage of blood spinal cord barrier [47,48]. Findings reveal that preliminary microvascular reactions in the spinal cord associated with t-SCI result into impairment of endothelial cell of the spinal cord, which eventually progresses to structural and functional loss of blood spinal cord barrier [49]. This barrier when destructed modifies the microenvironment of the spinal cord and results in infiltration of neutrophils and macrophages, thereby leading to the death of cells, inflammation, and long-term neurological malfunction [50]. Lowered blood spinal cord barrier damage exhibits marked neuroprotection after t-SCI episode [51]. Edema after the t-SCI event is a result of extravagant spinal cord water content in the intracellular and extracellular voids, which are often due to inflammation, trauma, and ischemia [52]. Spinal cord edema may either induce cytotoxicity or result in shock due to severe vasodilation. Excessive edema during t-SCI may damage the tight junction leading to exudation from blood vessels and resulting in intense vasodilation [53]. Altogether, edema in the spinal cord can aggravate damage of the blood spinal cord barrier and worsen the condition [52]. An increased spinal cord water content and EB effusion in t-SCI-induced rats were revealed in our investigation, exhibiting edema and blood spinal cord barrier breakdown. Administration of Eux (60 mg/kg) in t-SCI-induced rats significantly normalized the spinal cord water content and EB effusion values almost to normal, compared to that in the t-SCI animals.

The spinal cord is an organ that is protected by the blood–brain barrier, which makes it inaccessible to antibodies and associated immunomodulatory reactants. On damage during t-SCI, this barrier is damaged and breached resulting in a massive inflammatory response [54]. Interleukins, tissue necrotic factor-α, and inflammatory reactants are activated to ensure and help rapid repair of the spinal cord. Increased activation and release of these inflammatory mediators may harm normal cells [55]. Hence, investigations to explore modulation and regulation of the inflammatory responses may create newer avenues in the t-SCI treatment. Our results demonstrated that Eux (60 mg/kg) markedly lowered the inflammatory mediators, TNF-α, IL-6, IL-12, and IL-1β, thereby inducing an anti-inflammatory effect in t-SCI affected rats. Reports have demonstrated that secondary damages during t-SCI are associated with oxidation, indicated by the presence of oxidative stress markers such as MDA, SOD, GSH, and GSH-PX [56,57,58]. In our investigation, we inferred that Eux (60 mg/kg) markedly reduced the elevated MDA levels, whereas the reduced SOD, GSH, and GSH-PX levels were elevated in comparison to those in t-SCI-induced rats. The findings are as per those observed by Li et al. [32].

The NF-κB signaling cascade presents a key involvement in the initiation and progression of SCI and is linked with resulting responses. Inflammatory responses are known to phosphorylate IκB and activate the p65 subunit which then undergoes nuclear translocation [59]. Activated p65 triggers activation of IL-1β and TNF-α, which further stimulates NF-κB cascade. One of the key targets of a widely available mammalian MAPK cascade is NF-κB which is involved in the growth, multiplication, and death of cells [60]. The MAPK cascade is known to involve kinases that are dependent on hormones, cytokines, proteins, and miscellaneous growth factors. The initiation and progression of inflammatory responses is influenced by the modulation of the MAPK signaling cascade [61]. The MAPK stimulation triggers enhanced generation of TNF-α, IL-6, IL-12, and IL-1β, thereby producing severe inflammation coupled with immune reactions [62]. Our study depicted that Eux (60 mg/kg) markedly reduced the elevated TLR4 and NF-κB levels and triggered the p-p38 MAPK cascade in t-SCI-induced rats.

Stromal cells of bone marrow differentiate into neuronal and glial cells by the utilization of RANKL. Though the mechanism needs deeper understanding, this may involve the following mechanisms, (i) activation of RANK by RANKL binding, (ii) activation of NF-κB by the interaction between RANK and factors associated with TNF receptors, (iii) translocation of cytoplasmic NF-κB to the nucleus to link with kB onto specific genes thereby resulting into target gene transcription, and (iv) upregulation of transcription factors involved into the development of neurons which are genetically enhanced or lowered to regulate the signals which target the neural cells transduction. These further trigger neural cell development by stromal cells of bone marrow [63,64]. The present investigation exhibited a significant reduction in the t-SCI-mediated RANKL expression after Eux (60 mg/kg) treatment, which is elevated in a t-SCI event.

Cytokines and several growth factors released under stress conditions are known to trigger the Akt release, which is a midpoint of cellular signal transduction cascade, and a fundamental PI3K downstream signaling molecule [21]. Akt is critically involved in neuronal growth, development, multiplication, synaptic plasticity, and other major functions of the cells [65]. Earlier investigations have revealed that Akt is involved in the propagation of pain signals peripherally as well as centrally [66,67]. Blocking this PI3K/Akt signaling cascade induces a substantial antinociceptive effect [68]. Also, the widespread distribution of p-Akt in the dorsal horn region occurs after damage to the peripheral neurons [69]. Akt signaling cascade is critically involved in maintaining the plasticity of neurons damaged during the t-SCI challenge [70]. The Akt signaling cascade is known to be linked with the generation and propagation of neurological pain by its stimulatory effect on nociceptive perceptible neurons [71]. In our investigation, the results exhibited a marked suppression of caspase-3 and enhancement in the expression of PI3K and p-Akt in rats treated with Eux (60 mg/kg) after t-SCI induction, compared to the Eux untreated rats. Also, p38 expression was remarkably lowered in rats after treatment with Eux (60 mg/kg) in T-SCI-induced rats. The findings demonstrate that the PI3K/Akt signaling cascade and p38 modulation may be implicated in the antiapoptotic mechanism of Eux in a t-SCI event.

## 5 Conclusion

Altogether, Eux corrected the damage caused due to t-SCI and attenuated the progressive deterioration resulted after t-SCI event in experimental rats. The effects of Eux can be attributed to the correction of the levels of inflammatory and oxidative stress markers in the spinal cord. Eux resulted in reduction of TNF-α, IL-6, IL-12, and IL-1β level, thereby inducing an anti-inflammatory effect in t-SCI affected rats. Normalization of MDA, SOD, GSH, and GSH-PX levels in the spinal cord was exhibited by Eux treatment. The enhanced TLR4 and NF-κB levels were significantly lowered with Eux administration. Findings revealed that Eux triggered the p-p38 MAPK cascade in t-SCI-induced rats. Eux modulated the PI3K/Akt signaling cascade, thereby correcting the post-t-SCI damage in experimental rats. The findings, thus, advocate the protective effect of Eux in t-SCI induced in mice.
